# Metabolite Adjustments in Drought Tolerant and Sensitive Soybean Genotypes in Response to Water Stress

**DOI:** 10.1371/journal.pone.0038554

**Published:** 2012-06-07

**Authors:** Sonia Silvente, Anatoly P. Sobolev, Miguel Lara

**Affiliations:** 1 Centro de Ciencias Genómicas, Universidad Nacional Autónoma de México, Cuernavaca, Morelos, México; 2 Istituto di Metodologie Chimiche, Laboratorio di Risonanza Magnetica “Annalaura Segre”, CNR, Monterotondo, Rome, Italy; Purdue University, United States of America

## Abstract

Soybean (*Glycine max* L.) is an important source of protein for human and animal nutrition, as well as a major source of vegetable oil. The soybean crop requires adequate water all through its growth period to attain its yield potential, and the lack of soil moisture at critical stages of growth profoundly impacts the productivity. In this study, utilizing ^1^H NMR-based metabolite analysis combined with the physiological studies we assessed the effects of short-term water stress on overall growth, nitrogen fixation, ureide and proline dynamics, as well as metabolic changes in drought tolerant (NA5009RG) and sensitive (DM50048) genotypes of soybean in order to elucidate metabolite adjustments in relation to the physiological responses in the nitrogen-fixing plants towards water limitation. The results of our analysis demonstrated critical differences in physiological responses between these two genotypes, and identified the metabolic pathways that are affected by short-term water limitation in soybean plants. Metabolic changes in response to drought conditions highlighted pools of metabolites that play a role in the adjustment of metabolism and physiology of the soybean varieties to meet drought effects.

## Introduction

Soybean (*Glycine max*) is one of the most important grain legumes. It represents not only an essential source of protein, oil and micronutrients in human and animal diets, but is also an attractive crop for the production of biodiesel [1]. Soybean growth is affected by unfavorable environmental factors such as extreme temperatures, drought, nutrient deficiency and soil acidity, which form major constraints for soybean crop production.

Soybean plants form root nodule symbiosis with nitrogen-fixing bradyrhizobia, thus rendering the plant independent of N fertilizers. Nodulation and symbiotic nitrogen fixation have long been recognized as being sensitive to environmental stresses, particularly drought [2]. Water stress reduces nitrogen fixation as a result of a decrease in photosynthate supply [3] or a reduction in the O_2_ flux into the nodule as well as through overloading nodules with nitrogenous compounds [4]–[7].

Some of the most important responses of a plant against drought stress are associated with the accumulation of minerals [8] and the enhanced synthesis of osmoprotectants, or compatible solutes, which are part of normal metabolism. The accumulation of these compounds helps the stressed cells in water retention [9] and in the maintenance of the structural integrity of the cell membranes [10].

The types of osmoprotectant metabolites and their relative contribution in lowering the osmotic potential differ greatly among plant species. Osmotic adjustment has been reported in legumes with a high tolerance to water stress [11], [12]. Metabolic adjustments in response to the adverse environmental conditions may highlight pools of metabolites that play important roles in metabolism and physiology and may indicate which pathways have been perturbed by the stress.

Nuclear magnetic resonance (NMR) spectroscopy can be used to monitor and quantify the degree of metabolic impact induced by drought or other environmental disturbances [13], [14], since NMR can bring “high-throughput” spectroscopic/structural information on a wide range of metabolites simultaneously with high analytical precision. One of its main advantages is that it avoids biases against various classes of compounds. Molecular identification is easy and straightforward as it can be deduced from the NMR spectrum of the mixture itself by means of 1D and 2D experiments, standard additions and by comparison with database of standard compounds.

In the present investigation, ^1^H NMR-based metabolic profiling combined with the physiological studies were conducted in two genotypes of soybean differing in their tolerance to drought in order to elucidate metabolite adjustments in relation to the physiological responses in the nitrogen-fixing plants towards water stress. To our knowledge this is the first report on metabolite profiling in soybean under drought stress. NMR based metabolic profiling approach [15], [16] adopted in this study enabled the identification and quantification of a number of metabolites belonging to various classes of compounds from the crude extracts, without involving any separation step.

**Figure 1:**
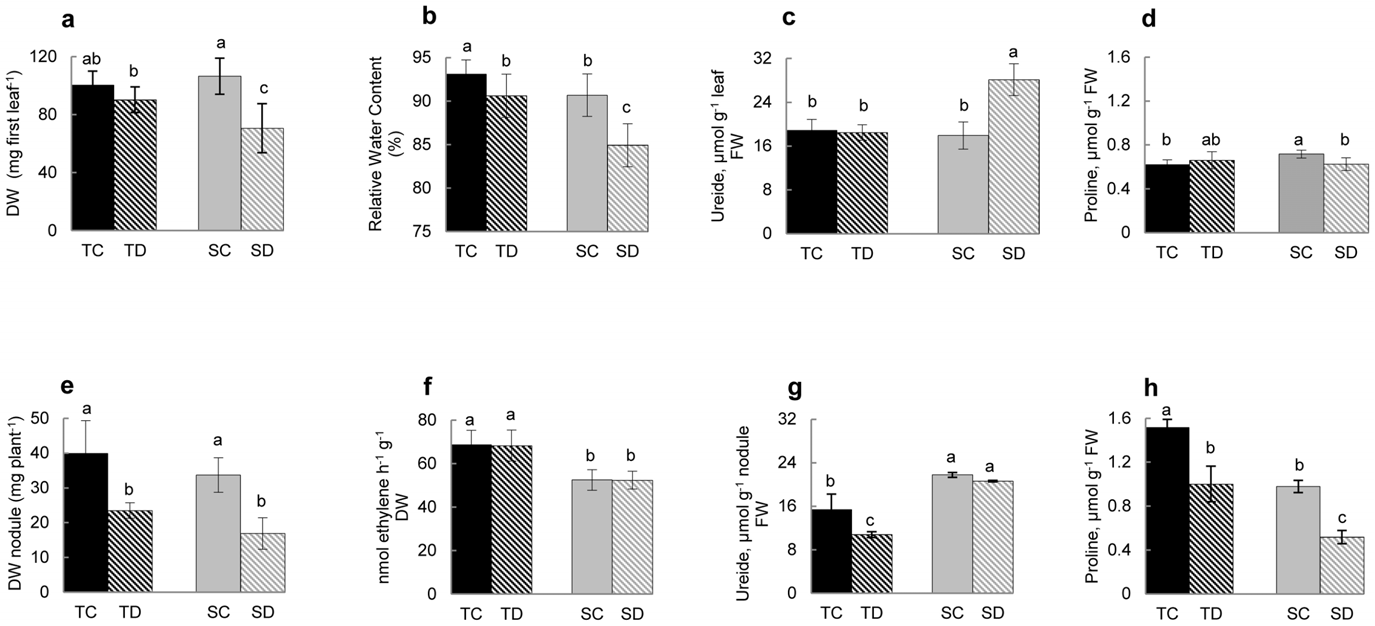
Responses of drought tolerant and sensitive varieties of soybean towards short-term water stress. (a–d) trifoliate leaves and (e–h) nodules. (a, e) dry weight (DW); (b) relative water content (RWC), (f) nitrogenase activity, (c, g) ureide levels and (d, h) proline levels in 17-day-old plants subjected to 10 days water sufficient and deficient conditions. TC: Tolerant genotype Control; TD: Tolerant subjected to Drought, SC: Sensitive genotype Control; SD: Sensitive subjected to Drought. Data represent the mean ± standard deviation (SD) of five to 15 replicates. Different letters at the top of each bar indicate significant differences at *P*<0.01.

## Results

The two soybean cultivars used in the present study were categorized at CIAP-INTA (Centro de Investigaciones Agropecuarias – Instituto Nacional de Tecnología Agropecuaria, Argentina) as tolerant (NA5009RG) and sensitive (DM50048) to drought stress based on their ability to maintain relative water content (RWC) and growth, and withstand oxidative stress through the modulation of cellular malondialdehide (MDA) levels. The drought tolerant line maintained higher RWC and showed greater ability to withstand oxidative damage owing to lower production of MDA, and exhibited sustained growth at reduced soil moisture conditions (Dr. Celina Luna, personal communication). In the present study, a comparison of these two soybean drought tolerant and sensitive genotypes was undertaken to determine the differences in their metabolic profiles/responses during water stress. Drought was imposed on nodulated soybean plants 17 days after inoculation by withholding water during 10 days, and physiological characteristics such as dry weight (DW), relative water content (RWC), chlorophyll and nitrogenase activity (ARA), and metabolite profiles were analyzed in order to establish the effect of water stress on these plant parameters (Figure 1). The results of the study showed that the water stress produced varied effects on leaf and nodule metabolism in both drought tolerant and sensitive soybean varieties. Under the drought condition imposed, both RWC and DW of the leaves showed a reduction, more remarkably in the sensitive variety (a decrease of about 10% in RWC and 42% in DW in the sensitive genotype as compared to 9% and 15%, respectively, in the tolerant one; Figure 1a, b). In addition, water stress affected proline and ureide contents in leaves of the sensitive variety but not in the tolerant genotype (Figure 1c, d). On the other hand, no effect of drought stress was observed on chlorophyll content as well as chlorophyll *a/b* ratio in both the genotypes (data not shown).

In case of nodules, in both genotypes, water stress conditions even for 10 days resulted in a dramatic reduction in DW, even though the nitrogenase activity per unit DW remained unaffected (Figure 1e, f). Similar to DW, drought conditions also caused fall in both proline and ureide contents in the tolerant variety (Figure 1g, h). In contrast, in the sensitive genotype, only proline declined while ureide content remained unchanged in the nodules as compared to that in well watered plants.

### Comparison of the metabolite profiles of leaf and nodule tissues of tolerant and sensitive varieties subjected to drought

**Figure 2:**
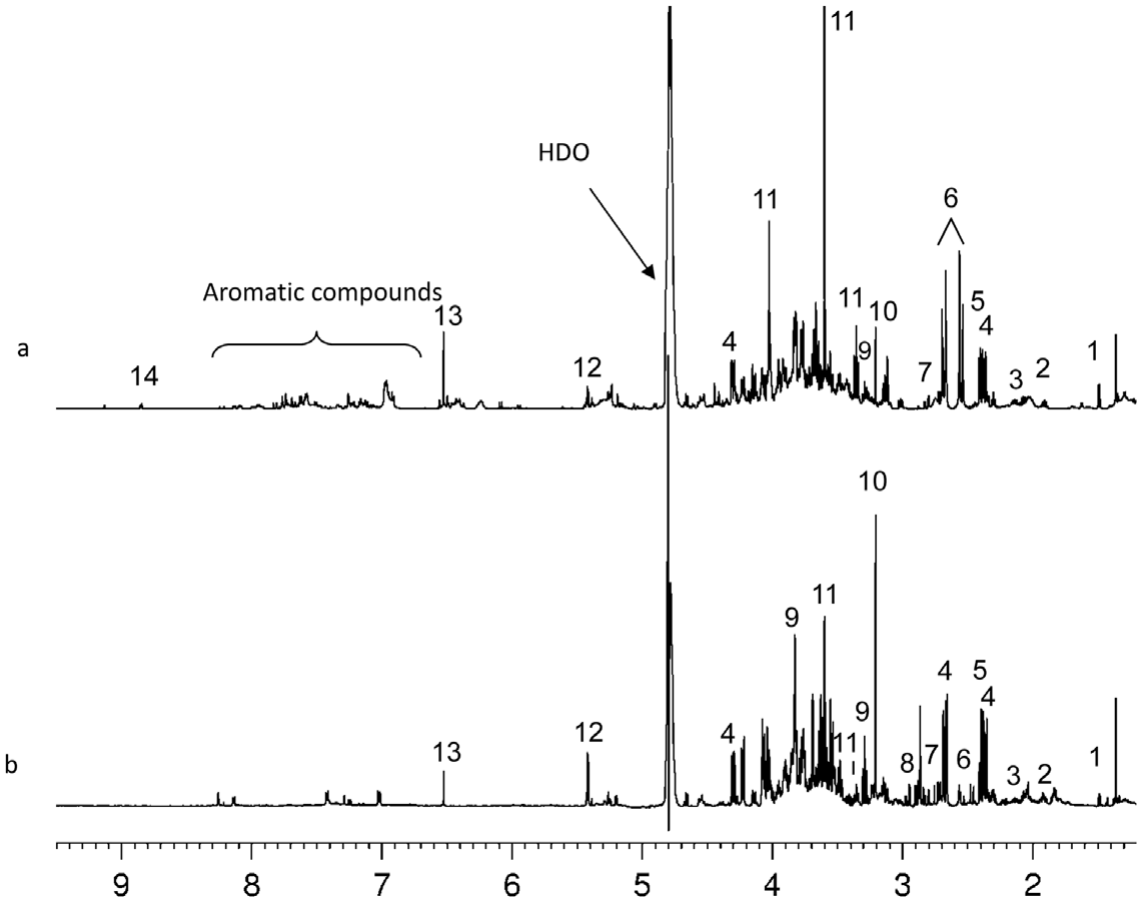
^1^H NMR spectra of (a) leaves and (b) nodules of water-soluble extracts from well-watered tolerant soybean plants. Assignments: 1, alanine; 2, GABA; 3, glutamine; 4, malic acid; 5, succinic acid; 6, citric acid; 7, aspartate; 8, asparagine; 9, myo-inositol; 10, choline; 11, pinitol; 12, sucrose; 13, fumaric acid; 14, trigonelline. HDO: deuterated water.

Comparison of the ^1^H NMR spectra revealed no major qualitative differences in the metabolites between leaf and nodule tissues except for minor aromatic compounds (Figure 2 and Table 1). As an example, a typical ^1^H NMR spectra of leaf and nodule extracts of well watered tolerant soybean plants together with the assignment of the most abundant metabolites (amino acids, sugars, organic acids) are shown in the Figures 2a and 2b, respectively. Among the resonances in the 9.5 – 7 ppm region, characteristic for aromatic and heteroaromatic compounds, only trigonelline was assigned in leaves extracts. On the other hand, asparagine was detected exclusively in nodules.

**Table 1:** List of variables used in statistical analysis for leaves and nodules samples.

Compound	Abbreviation	^1^H Chem. shift, ppm
**Ala**		1.49
**GABA**		1.91
**Gln**		2.14
**Malic A**	MA	2.38
**Succinic A**	SU	2.41
**2-Oxoglutaric A**	Ox	2.45
**Citric A**	CI	2.53
**Asp**		2.83
Asn (*)		2.98
**Choline**	CH	3.21
**Myo-inositol**	MI	3.29
**Pinitol**	PI	3.36
**Allantoin**	AL	5.39
**Sucrose**	SUCR	5.42
**Fumaric A**	FU	6.52
Trigonelline (**)	Trig	8.85

Note: (*) nodules, (**) leaves.

**Figure 3:**
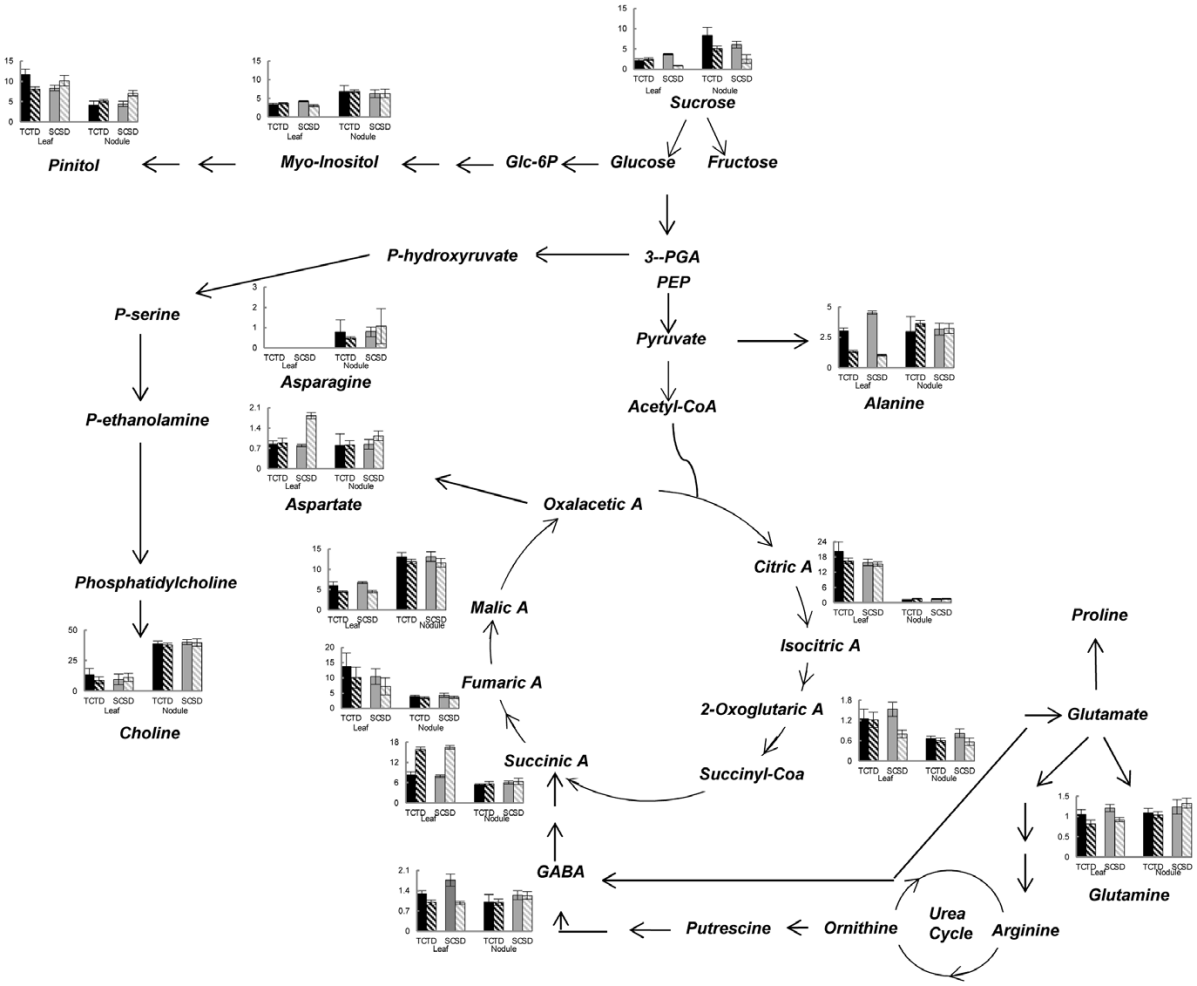
Schematic representation of the selected metabolic pathways affected by drought in two soybean genotypes contrasting in sensitivity/tolerance to water stress. Histograms represent relative changes in the level of the metabolites (arbitrary units) in trifoliate leaves and nodules in the plants subjected to water stress. Values are presented as the mean ± standard deviation (SD) of nine independent biological determinations. TC: Tolerant control; TD: Tolerant Drought, SC: Sensitive Control; SD: Sensitive Drought.

Basically, regardless of the genotype (tolerant or sensitive plants) or watering conditions, all the ^1^H NMR spectra of extracts from the same type of tissues (leaves or nodules) share the same signals, although their relative intensity is variable. The intensity of selected signals (Table 1 and Materials and Methods section) was used to calculate the relative molecular abundance of about 15 assigned metabolites. On the other hand, the assignment of minor components was hindered by the scarcity of data on these metabolic compounds in literature. Although the number of compounds identified by NMR is limited, the NMR spectra indeed gave a good picture of what really is present in the plant extracts examined. Results of the study demonstrated that water stress induces several changes in various metabolic pathways in both genotypes; the effect being more pronounced in the leaves than in nodules (Figure 3 and Tables 2,3,4).

Under drought conditions, levels of the individual sugars varied considerably among the genotypes: for example, sucrose and myo-inositol levels in the leaves decreased drastically in the sensitive genotype, but no significant changes were observed in the tolerant variety. In contrast, in the leaves of the sensitive genotype, pinitol levels increased under drought while it decreased in the tolerant one. In nodules, however, sucrose content decreased in drought in both varieties while pinitol levels increased. Myo-inositol content, on the other hand, did not alter in the nodules of the both varieties when the water stress was imposed.

Individual organic acids that mainly contributed to the differences in total organic acids under drought were 2-oxoglutaric acid, succinic acid and malic acid. Of these three, only succinic acid levels rose while malic acid content decreased in the leaves of drought stressed plants as compared to well-watered plants in both the genotypes, with no significant changes in nodules. 2-oxoglutaric acid, on the other hand, showed downward trend only in the leaves of sensitive variety.

With regard to the free amino acids in the leaves under drought, the contents of alanine and glutamine decreased in both the genotypes. On the other hand, GABA declined only in the tolerant one, whereas aspartate levels increased in the sensitive genotype. In contrast, no significant differences were observed in the amino acid contents in the nodules of both cultivars under control and stress conditions (Figure 3 and Table 4).

**Table 2:** Two factors ANOVA with a 2×2 between groups design (drought tolerant vs. sensitive plants, well-watered vs. drought stressed plants), leaves samples.

Metabolites	Control vs Stressed		Tolerant vs Sensitive		Interaction	
	F	p-level	F	p-level	F	p-level
**Ala**	2207.38	0.00E+00	121.02	2.09E-12	274.79	2.89E-17
**Gln**	71.66	1.13E-09	17.84	1.86E-04	0.96	3.36E-01
**Asp**	199.14	2.73E-15	134.92	5.15E-13	171.96	2.04E-14
**GABA**	159.3	5.73E-14	27.94	8.64E-06	34.04	1.75E-06
**MA**	112.1	5.50E-12	3.99	5.45E-02	3.93	5.59E-02
**CI**	9.21	4.75E-03	14.66	5.65E-04	4.64	3.88E-02
**SU**	1588.19	7.58E-29	0.24	6.27E-01	5.45	2.59E-02
**OX**	27.56	9.60E-06	0.92	3.45E-01	23.12	3.46E-05
**FU**	9.38	4.43E-03	7.62	9.47E-03	0.03	8.54E-01
**MI**	31.17	3.64E-06	1.56	2.20E-01	94.60	4.45E-11
**PI**	6.77	1.39E-02	4.21	4.84E-02	62.40	5.15E-09
**SUCR**	181.79	9.59E-15	0.03	8.72E-01	295.46	1.02E-17
**CH**	1.48	2.32E-01	0.33	5.71E-01	5.20	2.93E-02
**AL**	3.07	8.91E-02	49.18	6.00E-08	121.49	1.99E-12
**Trig**	11.57	1.82E-03	9.06	5.06E-03	0.01	9.07E-01

**Table 3:** ANOVA on single groups, two types of grouping (control vs stressed, and tollerant vs sensitive) leave samples.

	Control vs Stressed				Tolerant vs Sensitive			
	Tolerant		Sensitive		Control		Stressed	
Metabolite	LTC vs LTD		LSC vs LSD		LTC vs LSC		LTD vs LSD	
	F	p-level	F	p-level	F	p-level	F	p-level
**Ala**	291.72	1.10E-11	4862.95	2.60E-21	220.19	9.00E-11	56.94	1.20E-06
**Gln**	20.73	0.00033	68.84	3.40E-07	10.03	0.00598	8.08	0.01175
**Asp**	0.34	0.56602	675.71	1.60E-14	2.07	0.16953	209.71	1.30E-10
**GABA**	36.97	1.60E-05	123.69	6.10E-09	36.95	1.60E-05	0.46	0.50727
**MA**	22.61	0.00022	217.86	9.70E-11	4.69	0.04581	0.00	0.98729
**CI**	7.57	0.01422	1.76	0.20349	10.02	0.00599	6.56	0.02094
**SU**	477.46	2.40E-13	1691.62	1.20E-17	1.50	0.23907	4.63	0.04702
**Ox**	0.07	0.79541	84.41	8.80E-08	5.65	0.03023	24.13	0.00016
**FU**	3.82	0.06824	6.67	0.02006	3.82	0.06824	3.83	0.06796
**MI**	7.55	0.01432	135.87	3.10E-09	67.38	4.00E-07	32.48	3.30E-05
**PI**	56.65	1.20E-06	13.67	0.00195	46.31	4.20E-06	18.37	0.00057
**SUCR**	3.84	0.06779	2236.63	1.30E-18	108.64	1.50E-08	226.12	7.40E-11
**CH**	5.85	0.02785	0.59	0.45256	2.98	0.1037	2.31	0.14815
**AL**	66.89	4.20E-07	55.08	1.40E-06	8.09	0.01171	161.52	8.90E-10
**TRIG**	4.41	0.05201	7.97	0.01223	3.87	0.06674	5.32	0.03473

**Table 4:** Two factors ANOVA with a 2×2 between groups design (drought tolerant vs. sensitive plants, well-watered vs. drought stressed plants), nodules samples.

Metabolite	Control vs Stressed		Tolerant vs Sensitive		Interaction	
	F	p-level	F	p-level	F	p-level
**Ala**	2.70	1.12E-01	0.27	6.05E-01	2.02	1.67E-01
**Gln**	0.37	5.51E-01	18.12	2.38E-04	1.79	1.92E-01
**Asp**	3.81	6.16E-02	4.38	4.63E-02	2.74	1.10E-01
**GABA**	0.03	8.74E-01	15.28	5.92E-04	0.02	8.80E-01
**MA**	12.52	1.54E-03	0.25	6.22E-01	0.39	5.38E-01
**CI**	22.78	6.13E-05	1.72	2.01E-01	4.62	4.11E-02
**SU**	3.86	6.04E-02	7.80	9.69E-03	0.05	8.27E-01
**OX**	16.31	4.22E-04	2.63	1.17E-01	8.04	8.74E-03
**FU**	10.30	3.52E-03	2.39	1.34E-01	0.84	3.67E-01
**MI**	0.03	8.57E-01	2.07	1.62E-01	0.00	9.65E-01
**PI**	52.88	1.01E-07	20.71	1.10E-04	10.04	3.90E-03
**SUCR**	52.81	1.02E-07	27.54	1.75E-05	0.00	9.49E-01
**CH**	0.44	5.14E-01	3.71	6.51E-02	0.12	7.37E-01
**AL**	4.32	4.77E-02	23.31	5.29E-05	1.92	1.78E-01
**Asn**	0.01	9.42E-01	2.27	1.44E-01	2.12	1.57E-01

Principal component analysis (PCA) is one of the most popular explorative methods used to reduce multivariate data complexity. This is a method of choice for identifying patterns, and expressing data in ways that highlight similarities and differences between samples [17]. In our study, PCA was applied to ^1^H NMR spectral data of control and stressed leaves and nodules derived from two soybeans genotypes with varied tolerance to drought, in order to authenticate the differences between the metabolic profiles of the control and stressed tissues statistically and to identify the main metabolites responsible for the differences.

**Figure 4:**
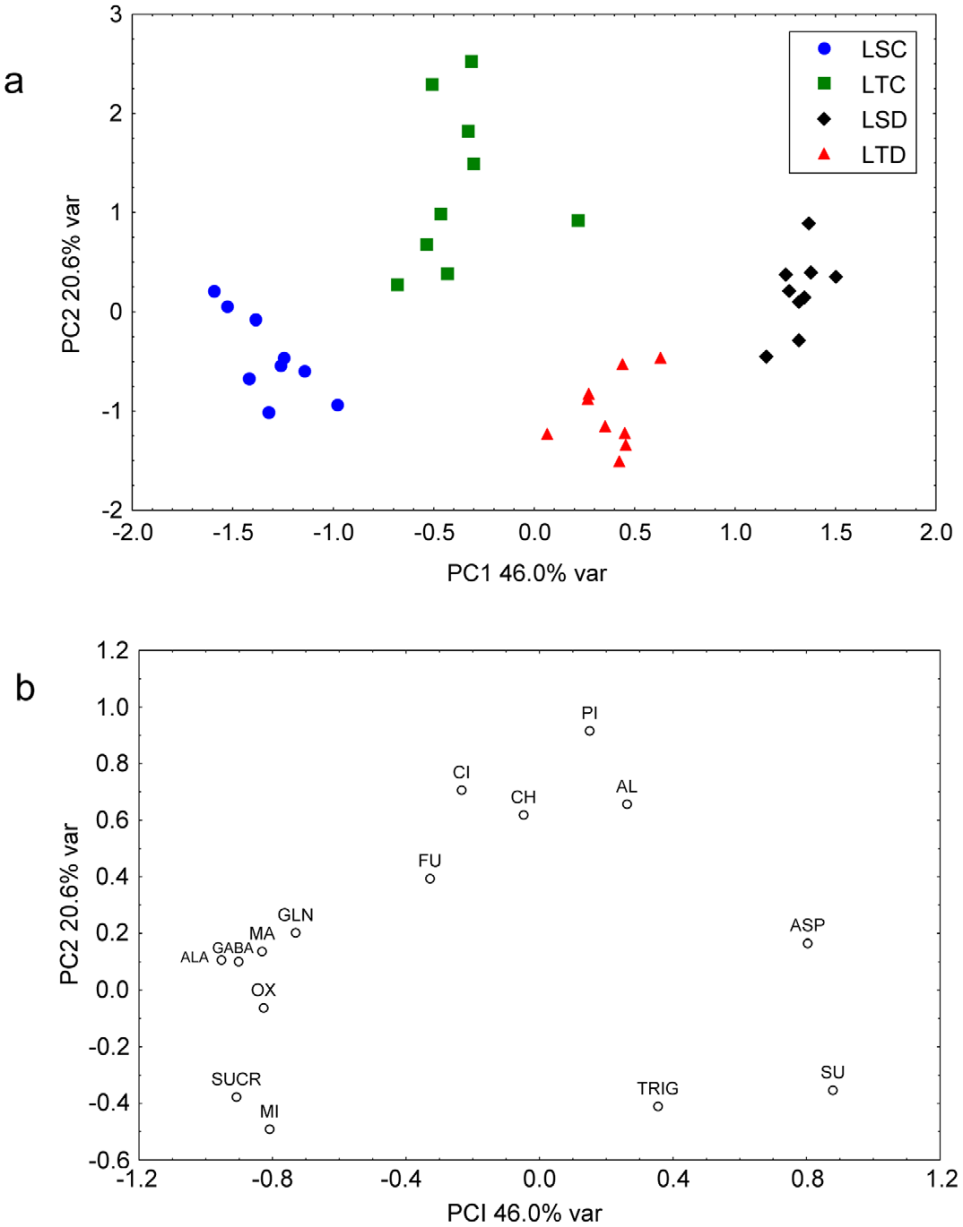
Principal component analysis (PCA) of 15 metabolites in the leaves from the plants grown under water sufficient and deficient conditions. Score (a) and loading plot (b) of soybean leaf samples. LTC: Leaf Tolerant Control, LTD: Leaf Tolerant Drought, LSC: Leaf Sensitive Control, LSD: Leaf Sensitive Drought.

A scores scatter plot of the first two PCs obtained considering all ^1^H NMR data derived from the leaves shows a good separation of all four groups (LTC: leaf tolerant control, LTD: leaf tolerant drought, LSC: leaf sensitive control and LSD: leaf sensitive drought) along PC1 axis (Figure 4a). It seems that this separation is due to the treatment (control vs stressed) with further separation between stressed sensitive and stressed tolerant genotypes. The greatest separation along PC1 is between LSC and LSD groups whereas the separation between LTC and LTD is less apparent along PC1 axis, but noticeable along PC2 (see arrows on Figure 4a). This behaviour of the data evidences a markedly more profound metabolic impact of drought stress on sensitive plants with respect to tolerant ones. The separation between leaf samples of well watered and stressed plants along PC1 axis seemed to be mainly attributable to aspartate, succinic acid, sucrose, malic acid, alanine, GABA, myo-inositol and 2-oxoglutaric acid as shown in the complementary PCA loading plot (Figure 4b). In case of the tolerant genotype, leaf samples of control and stressed plants are well separated along PC2 due to the metabolites pinitol, citric acid, choline, and allantoin.

A comparison of metabolite mean levels between LSC and LSD samples (sensitive plants) and between LTC and LTD ones (tolerant plants) was performed using ANOVA (Table 3). ANOVA results confirm the observations obtained with PCA. In fact, the levels of 11 out of 15 metabolites was significantly changed in sensitive plants upon the application of drought stress, whereas lesser number of metabolites (7 out of 15) were influenced by the stress in tolerant plants. Considering drought treatment and genotype as two independant factors and possible interaction between them, ANOVA has been applied using a 2×2 between group design (Table 2). This approach was aimed to give a statistical measure of significance for each factor and interaction between them for each variable. The criterion of statistically significant difference between the mean values was p-level less than 0.01. For 8 variables (alanine, aspartate, GABA, 2-oxoglutaric acid, myo-inositol, pinitol, sucrose, allantoin) out of 15 the interaction between two factors was found to be statistically significant, evidencing different responses of sensitive and tolerant plants to the drought stress on the molecular level. In fact, for aspartate, 2-oxoglutaric acid, myo-inositol, pinitol, sucrose and allantoin the trends of changes upon the application of drought stress is opposite in tolerant and sensitive plants. For example, we can see that in tolerant plant samples, the level of pinitol is higher in control than in water stressed plants, while this is reversed when the sensitive plants were subjected to water stress. It is seems that the idea of considering treatment and genotype as independant factors is not adequate, as the stress produces different results in tolerant and sensitive samples.

**Figure 5:**
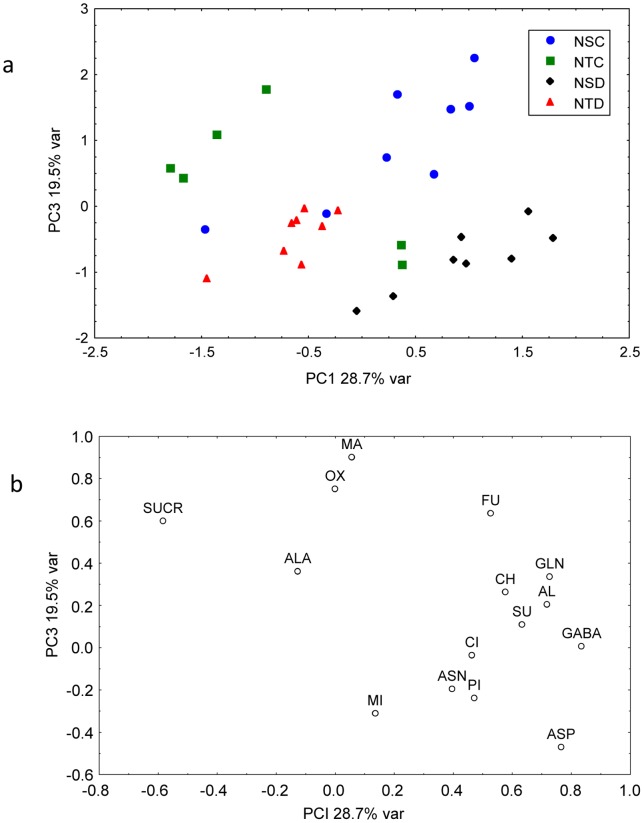
Principal component analysis (PCA) of 15 metabolites in nodules from the plants subjected to water sufficient and deficient conditions. Score (a) and loading plot (b) of soybean nodule samples. NTC: Nodule Tolerant Control, NTD: Nodule Tolerant Drought, NSC: Nodule Sensitive Control, NSD: Nodule Sensitive Drought.

In nodules, the PCA analysis (Figure 5a) showed that the first two PCs represented 48.2% of the initial variability contained in the original data. The scores plot exhibited separation between all four groups (NSC: Nodule Sensitive Control, NSD: Nodule Sensitive Drought, NTC: Nodule Tolerant Control and NTD: Nodule Tolerant Drought) when PC2 and PC1 were used as variables. It seems that with a few exceptions, the samples of tolerant plants are separated from sensitive ones along PC1, while control samples are separated from stressed ones along PC2. Plot of loadings (Figure 5b) show the variables responsible for this separation. The metabolites sucrose, aspartate, glutamine, GABA, allantoin, and succinic acid play a crucial role in the separation of tolerant from sensitive samples. On the other hand, the separation of controls from the stressed samples is due to the variations in the levels of malic acid, 2-oxoglutaric acid, fumaric acid, and sucrose.

The ANOVA analysis (Table 4) confirmed the same variables (that were identified by PCA) as statistically significant for the separation of groups. In addition to this, ANOVA revealed that the level of pinitol and citric acid is significantly different in control and stressed nodule tissues. It is notewothy, that only in the case of two metabolites (pinitol and 2-oxoglutaric acid) the interaction between genotype and drought treatment was significant.

## Discussion

In plants, the level of tolerance or sensitivity to water stress depends on the species and genotype, length and severity of water loss, as well as on the developmental stage. In *Aeluropus lagopoide*, Mohsenzadeh S, *et al* [18] found significant correlation between leaf relative water content (RWC) and relative growth rate, net photosynthesis rate, chlorophyll and proline contents. The leaf RWC directly reflects the water status of plants and it can be used to identify the genotypes tolerant to stress [19]. In our study, the highest level of RWC was found in the cv. NA5009RG (Figure 1b), and according to Rampino *et al*. [19] this cultivar could be defined as a genotype tolerant to water deficit. In addition, even under water limitation, this variety showed only a marginal reduction in leaf RWC as compared to the sensitive genotype (DM50048) where RWC was significantly affected. Moreover, our results showed a notable reduction in leaf DW only in the sensitive genotype (Figure 1b-a). The decrease in DW under drought in the sensitive soybean variety might be related to the depletion of sucrose in the leaves of this genotype (Figure 3). These results are in agreement with the findings of Reddy *et al*. [20] who reported that water stress inhibited dry mater production due to limitation of photosynthesis. Differences in the reduction in leaf DW under water stress has also been reported for different legume species: 78% in mungbean, 60 % in cowpea and 37% in peanut compared with the unstressed plants [21].

Despite of the reduction in the RWC and dry weight of the leaves of the sensitive genotype under drought, no parallel decrease in chlorophyll content was observed (data not shown). This result is in agreement with Ashraf and Iram [12] who also observed a lack of effect of drought on chlorophyll content, and suggested that it could be due to the mild moisture stress to which the experimental plants were exposed.

Enhanced tolerance of plants to low water availability is attributed to the accumulation of soluble sugars in water-stressed tissues [22], acting as osmoprotectants [23], [24]. In contrast to this, the results of the present study with the twenty seven-day old plants showed no enhanced accumulation of soluble sugars such as sucrose and myo-inositol in the leaves of both genotypes (Figure 3; indeed sugar content decreased in the sensitive variety), indicating that these sugars do not play an osmoprotectant role at least at the early stages of the plant growth. Similar findings have been reported for other legume species exposed to osmotic stress [25].

In legumes, pinitol is a common sugar alcohol and it has been described as a common osmoprotectant [11], [26]. Our results showed that the tolerant genotype has higher amounts of pinitol even under normal conditions as compared to the sensitive variety. On the other hand, pinitol synthesis was found to be enhanced in the sensitive genotype under water stress. Future work can only establish the exact role of pinitol in osmoprotection of soybean plants.

Accumulation of amino acids was suggested to aid stress tolerance in plants, through osmotic adjustment, detoxification of reactive oxygen species and by intracellular pH regulation [27], [28]. An equivalent role for most of the amino acids, detected in our experiments, seems unlikely because their content, with the exception of aspartic acid, tended to decrease or remain constant even under drought in both genotypes (Figure 3). These results are in agreement with the findings in *Phaseolus vulgaris* [29].

Proline, an imino acid, is widely regarded as a main osmoprotectant in water stress tolerance in plants. In the present study with the plants at the vegetative stage, we found that water stress does not trigger enhanced proline synthesis (Figure 1d, h). In contrast, when the drought treatment was imposed during the flowering stage, there was a considerable increase in proline levels in the leaves and nodules of both genotypes (data not shown). Increases in proline level were also observed in others varieties of soybean, when drought was imposed at the reproductive stage and the RWC was lower than the values observed in the present work [30], [31]. Fukutoku and Yamato [32] reported that in intact soybean leaves remarkable proline accumulation occurred only when water stress became severe and protein metabolism was disturbed. These results suggest that even under water stress, the stage of the plant and RWC of the leaves seem to be critical in promoting proline synthesis. Role of proline in stress tolerance remains controversial as some authors have reported high proline levels in the susceptible cultivars subjected to stress conditions [33], [34], while the others have observed the opposite trend [35]. It has been suggested that proline functions as an indicator of plant water status but not a measure of level of tolerance [36].

In both soybean cultivars, drought did not trigger the accumulation of organic acids excepting succinate as its concentration was doubled in the leaves of both genotypes after the imposition of drought. Sassi *et al*. [29] reported a decrease in the total amount of organic acids in the leaves of a sensitive line of bean plants subjected to osmotic stress.

Present study demonstrated that water stress has varied effects on the metabolic processes of leaves and nodules in the soybean cultivars tested; Nodule DW was more affected than the leaf, and in the nodules of both genotypes the decrease in DW mirrored the drought-induced decline in sucrose. However, drought didn't affect the nitrogenase activity. The lack of response of nitrogenase activity to drought in these soybeans cultivars is in contrast to earlier reports [37], [38], [7], and it perhaps reflects the level of intensity of the stress imposed in various studies. In several soybean cultivars, inhibition of nitrogen fixation under drought stress has been attributed to ureide accumulation in leaves [39]. In the present investigation, ureide accumulation in response to drought stress was observed only in the leaves of the sensitive genotype, but not in the tolerant variety (Figure 1). Since the nitrogenase activity was unaffected under drought stress in both the genotypes, it seems that ureide accumulation in the leaves does not have a feedback inhibitory effect on nitrogen fixation. Alamillo *et al*. [40] suggested that ureide accumulation and nitrogen fixation follow different kinetics and are probably not causally related.

In nodules, malic acid is the most abundant organic acid and is the main carbon substrate for bacteroid respiration and nitrogen fixation activity. It had been suggested that a decrease in nodule malic acid content under certain environmental conditions may lead to the inhibition of nitrogen fixation [3]. Also in the present work, genotype differences in malic acid content or interactions between genotypes and drought were found to be absent (Figure 3), a situation that is consistent with the results of ARA activity under stress condition (Figure 1).

The analysis of metabolites contributes to the understanding of stress biology of plants through the identification of the compounds and the part they play in acclimation or tolerance response. In the present study, metabolite fingerprinting and profiling based on ^1^H NMR spectra were used to analyze the similarities and differences among leaf and nodule samples obtained from two soybean genotypes with the aim of identifying markers useful for pinpointing water stress response. In this context the results of our study point to six metabolites in leaves (aspartate, 2-oxoglutaric acid, myo-inositol, pinitol, sucrose, allantoin) and two in nodule (2-oxoglutaric acid and pinitol) that were affected differentially in the genotypes when drought was imposed at the vegetative stage in the nodulated soybean plants. These data provide information that may, with further experimentation, allow elucidation of biochemical pathway underlying stress tolerance in soybean.

The results of the study demonstrated that a combination of ^1^H NMR and multivariate analyses allows comparisons of overall metabolite fingerprints and that this technique can be applied to conclusively identify differences that are due to stress or genotype. The differences under stress conditions between the two genotypes discussed above are reflected in the PCA models of metabolite content as well. PCA of the present study clearly demonstrated that the major variability in metabolites levels (associated with PC1 in PCA) is due to treatment (control vs stressed) in the case of leaves, while in the case of nodules the major variability is due to genetic makeup (tolerant vs sensitive). The phenomena observed in the case of nodules likely depends on the cumulative effects of plant-bacterial genotypes, specificity of interaction and the resultant symbiosis which in turn alters the metabolism outcome.

## Materials and Methods

### Plant materials and growth conditions

Seeds of the soybean genotypes (*Glycine max* L. Merr.), namely NA5009RG (drought tolerant) and DM50048 (drought sensitive) were obtained from CIAP-INTA (Centro de Investigación Agropecuaria-Instituto Nacional de Tecnología Agropecuaria), Argentina. Seeds were surface sterilized with 20% (v/v) commercial bleach, washed extensively with sterile-distilled water, and germinated on a sterile moist filter paper at 28°C, in the dark, for 3 days. Subsequently, the seedlings were transferred to vermiculite in pots and inoculated with 1 cm^3^ of *Bradyrhizobium japonicum* USDA110, and grown in a greenhouse at 26°C/19°C (day/night temperatures). Plants received nitrogen-free Summerfield nutrient solution [41] twice a week until the stress treatments were imposed.

Drought stress was imposed on 17-day old plants at the vegetative stage by withholding water supply for 10 days until soil water content reached 23% (0.230 g H_2_O g^−1^ dry soil). A set of well-watered plants served as a control. Measurements of dry weight, relative water content and nitrogen fixation, as well as sampling of the plant tissues (nodules and first trifoliate leaves) for chlorophyll, proline, ureide and metabolome analysis were performed at the end of the stress period.

The results presented are the means with standard deviations of five to 15 replicates. All data obtained was subjected to one-way analysis of variance (ANOVA), and the mean differences were compared by lowest standard deviations (LSD) test using the STATISTICA package for Windows (version 5.1, 1997). Comparisons with *P* values<0.01 were considered significantly different.

### Leaf relative water content (RWC)

RWC was measured according to Barrs and Weatherly [42]. Briefly, immediately after sampling, the leaves were weighted and then soaked overnight in distilled water at 4°C. After the cold incubation, the leaves were blotted dry and weighed prior to oven-drying at 80°C for 48 h. Subsequently, dry weight of the plant samples was determined. The leaf relative water content was calculated using the following formula: RWC  =  (FW – DW)/(TW-DW)) ×100, where FW is fresh weight, DW is dry weight, and TW is turgid weight (weight after the leaf was kept in distilled water for overnight).

### Nitrogen fixation

Nitrogenase activity was determined by acetylene reduction assay (ARA), [43].

### Estimation of ureide content

Concentration of ureides present in leaf and nodule cell-free extracts was measured using the colorimetric detection method of Vogels and Van Der Drift [44]. Allantoic acid dissolved at a concentration of 10 mM in water served as a standard for ureide estimation.

### Determination of proline

Samples of fresh plant tissues (0.5 g) were homogenized in 5 ml of 3% aqueous sulphosalicyclic acid and supernatant was collected after centrifugation. Two mL extract was reacted with 2 mL acid-nihydrin and 2 mL glacial acetic acid, and incubated for 1 h in a boiling water bath. The reaction was terminated by placing the test tubes on ice after which the reaction mixture was vigorously mixed with 2 ml of toluene. After warming to 25°C, proline present in the upper toluene layer was measured at 520 nm [45].

### Metabolite extraction and NMR analysis

Comparative metabolite profiling was performed in leaves and nodules derived from the drought tolerant and sensitive soybean genotypes subjected to water stress. First trifoliate expanded leaves and nodules were collected after 10 days of drought treatment, and immediately frozen in liquid nitrogen, lyophilized and stored at −80°C till they were subjected to NMR analysis.

Water-soluble extracts were derived from 20 mg of lyophilized tissues mixed with 0.9 mL of CH_3_CN:H_2_O (1∶1 v/v); Extracts were clarified by centrifugation at 10,000 rpm (5,600 g) for 7 min, and the supernatant obtained was filtered and lyophilized. The dry residue was dissolved in 0.7 mL of 400 mM D_2_O phosphate buffer (pD = 6.5) containing 1.0 mM of 3-(trimethylsilyl) propionic−2,2,3,3-d4 acid sodium salt (TSPA) and transferred into a standard 5 mm NMR tube. NMR spectra of the extracts were recorded at 300 K on a Bruker AVANCE AQS600 spectrometer operating at the proton frequency of 600.13 MHz and equipped with a Bruker multinuclear z-gradient inverse probe-head capable of producing gradients in the z-direction with the strength of 55.4 G/cm.

Proton spectra were referenced to the signals of TSPA methyl group at δ = 0.00 ppm in D_2_O phosphate buffer. The ^1^H spectra of the aqueous extracts were acquired by co-adding 512 transients with a recycle delay of 2.5 s and 32 K data points (acquisition time 40 min). The residual HDO signal was suppressed using a presaturation during the relaxation delay with a long single soft pulse. To avoid possible saturation effects, the experiment was carried out by using a 45° flip angle pulse of 8.0 μs 2D NMR experiments, namely ^1^H–^1^H TOCSY and ^1^H–^13^C HSQC, were performed using the same experimental conditions as previously reported by Sobolev *et al* [46].

Fifteen metabolites in leaves and nodules extracts were identified and used for statistical analysis, see Tables 1 and 2. Metabolites were assigned and identified using 2D experiments ^1^H–^1^H TOCSY and ^1^H–^13^C HSQC and by comparison with the literature data [47].

The signal heights of selected ^1^H resonances of water-soluble metabolites (Table 1) were measured with respect to the height of TSPA signal used as internal standard. The height of TSPA signal was normalized to 100. The obtained values (relative molecular abundances of selected metabolites) were used in statistical analysis.

### Statistical Analysis of NMR data

The statistical treatment of the NMR data was performed using the STATISTICA package for Windows (version 5.1, 1997). Two factors ANOVA has been performed with a 2 × 2 between groups design (drought tolerant vs. sensitive plants, well-watered vs. drought stressed plants). Principal component analysis (PCA) was performed using all 15 variables for leaves and nodules. Before the PCA analysis the variables were mean-centered and each variable was divided by its standard deviation (autoscaling). The effects and interactions represented in bold in the Tables 2,3,4 were statistically significant within the 99% confidence interval.
